# EHMTI-0225. Peripheral nerve stimulation in drug-resistant cranial neuralgias

**DOI:** 10.1186/1129-2377-15-S1-C6

**Published:** 2014-09-18

**Authors:** PE Bermejo, C del Pozo, E Parodi, JM Ahijado, P Rey

**Affiliations:** 1 Neurology, Hospital Puerta de Hierro, Majadahonda, Spain; 2 Pain Unit, Hospital Puerta de Hierro, Majadahonda, Spain

## Introduction

Cranial neuralgias are distinct, treatable syndromes which comprise one of the possible causes of facial pain. Although some prophylactic medications and techniques have been proposed as treatments, there are still many refractory patients and other therapeutic options are warranted. Peripheral nerve stimulation (PNS) has been proposed as a promising therapy for these patients.

## Aim

The aim of this study is to evaluate the efficacy and tolerability of PNS for the treatment of refractory cranial neuralgias.

## Methods

Twelve patients (3 men, 9 women, average age 52.8±12.0) suffering from different drug-resistant cranial neuralgia were enrolled and implanted with a neurostimulation device. Five suffered from occipital neuralgia, 3 had postherpetic neuralgia and 4 had trigeminal neuralgia. The primary endpoint was the reduction in Analogical Visual Scale (AVS). Patient satisfaction, side effects and reasons for discontinuation were also studied. Significance level was set at P<0.05.

## Results

Pain severity according to the AVS was reduced from 9.0±0.9 before PNS to 4.9±2.7 after treatment initiation. 58% of treated patients were satisfied or very satisfied with the procedure. The most common adverse event was persistent implant site pain and three patients required to be explanted due to inefficacy. There were not differences between different subgroups.

## Conclusions

PNS has been explored as a possible treatment option in selective drug-resistant cranial neuralgias and, according to our results, this technique may be effective, safe and well tolerated in treating them. More studies are warranted to confirm these results.

No conflict of interest.

